# Supplementary material for: Theorization regarding access to oral health care for Brazilian prisoners: A qualitative study
Source: PLoS One. 2025 Oct 31;20(10):e0335590. doi: 10.1371/journal.pone.0335590 (PMC12578342; doi:10.1371/journal.pone.0335590)
Supplement: Attachment — Submitted filename: Response to Reviewers.docx [file pone.0335590.s002.docx]

**Dear Editor,**

We are pleased to have the opportunity for reviewing our manuscript. We conducted a comprehensive review of our manuscript. If, following our improvement, you decide further data and new analysis necessary, we will proceed accordingly.

**ABSTRACT AND INTRODUCTION**

**Suggestion 1:** Grounded Data Theory. C. Change this term to Grounded Theory.

**Solution:** We sincerely value your significant input to enhancing our manuscript. Indeed, we were employing a term that does not conform to international standards. Consequently, we will replace the term "Grounded Data Theory" with "Grounded Theory" throughout the abstract and methods sections. We made changes to lines 28 and 115.

**Suggestion 2:** 16 men were interviewed, and analysis allowed the creation of the following… C. Do not begin a sentence with numbers. (line 29)

**Solution:** We enhance our writing to prevent such blunders. The revised sentence is: We conducted interviews with 16 men, and analysis allowed the creation of the following categories related to the patient (lines 29 and 30).

**Suggestion 3:** 16 men were interviewed, and analysis allowed the creation of the following categories related to the patient: 1) Ability to perceive 2) Ability to seek 3) Ability to reach 4) Ability to pay 5) Ability engage, and categories related to the service offered: 1) Approachability 2) Acceptability 3) Availability and accommodation 4) Affordability 5) Appropriateness. C. Here the authors refer to the units of analysis as categories, and yet, in the manuscript they are presented as codes. The authors need to make a methodological choice and decide which one to use. It is my view that, since they have used a Theoretical Framework do organize the analysis, since it comes with pre-established conceptual categories, this makes more coherent to use them as categories (Conceptual Framework for Patient-Centered Access to Health Care by Levesque et al.14). As yet for the codes, then what is called sub-codes could be held as codes, making it again coherent with the methodology used.

**Solution**: We appreciate the input and agree with the suggested proposals. Consequently, at the locations where the terms "codes" and "subcodes" were utilized, they were substituted with "categories" and "subcategories." The substitutions occurred on the subsequent lines:

- Lines 156 and 157: Table 1 indicates the categories, subcategories, and illustrative statements of Prisoners’ Abilities to access oral health care.

- Lines 159 and 160: Table 1. Categories, subcategories, and illustrative statements constituting Prisoners’ Abilities in accessing oral health care in the prison system”

- Table 1, first line: “Categories and Subcategories”

- Lines 198 and 199: Table 2 presents Institutional Barriers of the Prison System, with its categories, subcategories, and illustrative statements.
- Lines 201 and 202: Table 2 Categories, subcategories, and exemplifying statements integrating the Structural Barriers of the Prison System, which limits access to oral health care in the prison system.
- Table 2, line 1: “Categories and Subcategories”

**Additional Solution**: We enhanced the parts of the text where the expression "Categories" was previously employed.

- Lines 153 to 155: “After the coding stages of the transcribed qualitative material, the categories emerged from the approximation of the meanings produced by prisoners in the interviews conducted using the patient-centered access to healthcare conceptual framework.

**Suggestion 4:** Brazilian government formulate health police to improve health care in the prisons few years ago. C. It has formulated. Please, say more about what health police means, it is hard to follow here. Or would it be a health policy?

**Solution:** We appreciate feedback and acknowledge that the expression "health policy" may have impeded comprehension of the content. Consequently, we have enhanced the content and will incorporate a concise section with further details regarding the specified public policy. Consequently, the subsequent paragraph has been appended.

Lines 57 to 64: A national policy for oral health care within the Brazilian jail system was proposed. Initially, this support was the obligation of the States, which enacted initiatives within prisons without definitive national directives. In 2014, this support was incorporated into the Unified Health System (SUS) by a public policy known as the National Policy for Comprehensive Health Care for Individuals Deprived of Liberty in the Prison System. Subsequently, health initiatives and services were initiated for jailed individuals, encompassing preventive, curative, and rehabilitative aspects. The primary aim of this program is to guarantee access to healthcare within the jail environment. [12]

**Suggestion 5:** The aim of this study was theorizing the meanings and interactions engender by Brazilian men prisoners about access oral health care.
C. Access to oral health care.

**Solution:** Thank you for the feedback; there was also a translation error. Nevertheless, in light of the additional ideas below, we propose a revised aim.

- Lines 23 to 25: In view of this, the aim of our study was to formulate a theory concerning access to oral health and the influencing factors, grounded in the perspectives of incarcerated men.

- Lines 66 to 68: The aim of our study was to formulate a theory concerning access to oral health and the influencing factors, grounded in the perspectives of incarcerated men.

**Suggestion 6:** Ability engage. C. This probably means Ability to engage.

**Solution:** We appreciate the feedback and concur with the recommendation. We changed the expression "Ability engage" to "Ability to engage" at all instances where the error occurred (lines: 31, 147 and 192)

**Suggestion 7:** The aim of this study was theorizing the meanings and interactions engender by Brazilian men prisoners about access oral health care. C. This objective is a bit complicated. Theorizing the meanings and interactions do not necessarily contribute to a specific aim within the Health System, unless it is pointed, at least to some extent, to help explaining the main issues related to access to oral health care, which seems to be the main focus of this paper. It is my suggestion that the authors choose a more precise objective, away from this one that is too open.

**Solution:** We value the recommendation and present a more precise objective for what is written in our article. The revised phrasing of the objective has been incorporated at end of the introduction and inside the abstract. “The aim of our study was to formulate a theory concerning access to oral health and the influencing factors, grounded in the perspectives of incarcerated men”. (Lines 23 to 25 and Lines 66 to 68)

**METHODOLOGY**

**Suggestion 8:** The interviews took place from June/2023 to November/2023, when theoretical saturation occurred. C. Theoretical saturation needs to be explained and supported by a relevant citation.

**Solution:** We accept with your feedback. Thus, we refine the explanation of theoretical saturation and cite two references utilized to define it.

Lines 95 to 98: The interviews were conducted from June 2023 until November 2023, at which point theoretical saturation was achieved [15]. This point delineated all dimensions of the theoretical framework [16] that were elucidated. The most challenging aspect to understand was the ability to reach dental treatment within correctional facilities.

**Suggestion 9:** Data collection was performed through semi-structured audio-recorded interviews, conducted by a single interviewer (author: NSBZ)
C. conducted by only one interviewer.

**Solution:** We are glad to accept your suggestion, and we replaced the sentence with “Data were collected through semi-structured, audio-recorded interviews conducted by only one interviewer (author: NSBZ).” (Lines 98 to 99).

**RESULTS**

**Suggestion 10:** Ability to pay. The conditions for accessing hygiene products and dental services are facilitated when the family of the individual incarcerate has the ability to pay for these products. C. It is unclear if the prisoners can access dental services if they could afford it, because in the way it has been written, it sounds like they could do it. Can they access dental treatment in a private dental office?

**Solution:** We understand that a more detailed explanation was lacking regarding the possibility of incarcerated men in Brazil acquiring private services. Thus, we chose to improve the wording of the topic.

Line 184 – 188: The conditions for accessing hygiene products and oral heath care are facilitated when the families of the incarcerated men have the financial means to pay. In Brazilian reality, when a family has sufficient financial resources, it is possible to request police escort so that the person deprived of liberty can be taken to private offices outside the prison unit. Moreover, family members can send oral hygiene products to prisoners, provided they comply with the security rules of the prison unit.

**Suggestion 11:** Acceptability. It was understood that there is discriminatory treatment in relation to the prisoners originates from the discrediting of the need for dental care by the prison institution. C. This sentence is hard to follow. Maybe the authors want to claim that ‘the discriminatory treatment in relation to the prisoners originates from’ and it has emerged in the analysis.

**Solution:** We appreciate the feedback, and we agree that there may have been a lack of clarity. Thus, we present a new draft for the paragraph.

- Line 212 - 219: The incarcerated men believe the discriminatory treatment in relation to the prisoners originates from criminal police officers. It occurs when individuals request dental appointments, and their healthcare needs are overlooked or not communicated to the prison health team. The interviewees indicate that the absence of direct contact with healthcare professionals implies a need for mediation by the prison authorities for their care requirements. Within these procedures, criminal police officers utilize refusal of dental consultations as a punitive strategy or to decrease undesirable behavior among the incarcerated population. This obstacle is demonstrated by the neglect of written requests and the refusal of escort from the cell to the dentist's office.

**Suggestion 12:** For them, the criminal police use preventing consultations as a disciplinary punishment method. C. It is unclear to me if the preventing consultations or the denial to access preventing consultations is considered punishment method. It seems that not accessing preventing consultations would be the case, but it is unclear in this paragraph.

**Solution**: We concur with the observation, and this issue was addressed with the prior solution. We present a new draft for the paragraph.

- Line 212 - 219: The incarcerated men believe the discriminatory treatment in relation to the prisoners originates from criminal police officers. It occurs when individuals request dental appointments, and their healthcare needs are overlooked or not communicated to the prison health team. The interviewees indicate that the absence of direct contact with healthcare professionals implies a need for mediation by the prison authorities for their care requirements. Within these procedures, criminal police officers utilize refusal of dental consultations as a punitive strategy or to decrease undesirable behavior among the incarcerated population. This obstacle is demonstrated by the neglect of written requests and the refusal of escort from the cell to the dentist's office.

**DISCUSSION**

**Suggestion 13:** Faced to understand the meanings produced by prisoners regarding access to oral health care, the results of our results… C. I guess the authors wanted to say the results of our study.

**Solution**: We value the feedback and concur that a drafting error occurred in this part. We now provide a revised draft of the sentence.

- Line 249 to 251: To understand the meanings produced by prisoners regarding access to oral healthcare, the results of our study indicate that difficulties in accessing oral health in the prison system are predominantly attributed to state negligence.

**Suggestion 13:** The values and ideals of the professionals in prison health teams also influence their actions towards the prison population, impacting in the implementation and functioning of the health service. C. In which sense? This is unclear as well, as to how it could be detrimental or not. Please rewrite it, such that this becomes clear to the reader.

Solution: We value the feedback and have revised it for clarity as below.

- Line 328-334: This conduct is shaped by ideological beliefs and ethical considerations grounded in a conservative perspective that the prison setting confines individuals who ought to forfeit all their rights, not merely their liberty. This affects the dynamics between criminal police officers and inmates, as well as the operation of healthcare services. In this setting, still significantly influenced by biases, these references exacerbate the stigmatization of the incarcerated population and rationalize the denial of rights, including oral health care. As a result, this diminishes the quality of health care and obstructs the execution of governmental policies [41].

**CONCLUSION**

**Suggestion 14:** The meanings identified in the interviews allowed us to understand that barriers to accessing oral health have been mainly attributed to state negligence, which results in overcrowding, lack of health professionals and criminal police officers. Furthermore, the prison interactions to access health care promotes a micropolitics of access which generates inequity in oral health. C. The state negligence results in overcrowding, lack of health professionals and criminal police officers – this may well be truth. Yet, it is a statement that perhaps goes beyond the aim proposed in this study. It might be the case that this Conclusion be more to the point of oral health care, pointing to the reasons to lack of access within the prison system. Such reasons are well described and discussed, as well. It is understandable that overcrowding is a reason, of course, but in a growing violence environment, in developing countries where social injustice is prevalent, vulnerability becomes a consequence and brings with it crimes and convictions, augmenting the incarcerated population. Thus, it is hard to deal with such a huge problem in an oral health scientific paper. Though the authors do not necessarily have to confine within the realm of oral health all the time, in the CONCLUSION it is necessary to conclude within the limits proposed by this field; hence, there is a need to produce a concise section where the main findings are brought up again, along with proposals do be implemented. Then, the authors are also invited to produce suggestions that might be used within the system, and that could be carried out. They could also make a more intricate discussion around the legislation – especially in the sense of how many dentists should/could be employed by the system to attend such a large demand, as well.

Solution: We value the recommendation and concur that the revision of the Conclusion was essential. We present below a rewriting conclusion.

- Lines 341-356: The meanings produced in the interviews allowed us to understand that the barriers to accessing oral health have been attributed by incarcerated men, mainly, to governmental negligence. This notion stems from the chronic scenario of overcrowding, the shortage of oral health and public safety professionals, which does not seem to be adequately addressed by the government. These problems reinforce inequalities, create a micro-politics of access, and consequently perpetuate inequity in access to oral health care for the incarcerated population.

Our results, as well as the precarious oral health conditions of the prison population, indicate the need to strengthen government policies aimed at health in the prison environment and to address overcrowding. In the realm of public policies, once the legal framework is established, it is up to the government’s responsibility to promote compliance with the defined parameters and to continuously oversee their execution. The legislative definition stipulates that the quantity and working hours of dental surgeons should be appropriate with the incarcerate population, varying from 6 hours per week for up to 300 detainees to 30 hours per week for facilities housing over 1,700 inmates. Ultimately, our findings underscore the necessity to reevaluate incarceration as a punishment for minor offenses and to enhance the training of criminal police officers about human rights, particularly in relation to healthcare access.
