# Supplementary material for: Theorization regarding access to oral health care for Brazilian prisoners: A qualitative study
Source: PLoS One. 2025 Oct 31;20(10):e0335590. doi: 10.1371/journal.pone.0335590 (PMC12578342; doi:10.1371/journal.pone.0335590)
Supplement: Attachment — Submitted filename: Response_to_Reviewers_auresp_2.docx [file pone.0335590.s004.docx]

**Dear Editor,**

We are pleased to have the opportunity to review our manuscript. We conducted a comprehensive review of our manuscript. If, following our improvement, you decide further data and new analysis are necessary, we will proceed accordingly.

**ABSTRACT**

**Suggestion 1:** The codes produced in the analysis were interpreted based on the Patient-Centered Access Conceptual Framework. C. Categories, not codes.

**Solution:** We appreciate the consideration given to our text. This term need to have been substituted in the prior revision. We substituted codes with categories at line 28 of the abstract.

“The categories produced in this qualitative analysis using Grounded Theory were interpreted and organized based on the Patient-Centered Access Conceptual Framework.”

**DATA COLLECTION**

**Suggestion 2:** Page 5, L 110. Data were collected through semi-structured, audio-recorded interviews conducted by only one interviewer (author NSBZ), a woman dental surgeon with. C. A female dental surgeon.

**Solution:** We express our gratitude for the consideration of our text. We improve our writing, and the amended sentence is “Data were collected through semi-structured, audio-recorded interviews conducted by only one interviewer (author: NSBZ), a female dental surgeon with no previous contact with the interviewees but with a history of providing care in the prison unit wherein the interviews were conducted (lines 97 and 100).

**THEORETICAL REFERENCE**

**Suggestion 3:** Page 6

The conceptual framework for patient-centered access to health care proposed by Levesque et al. [16] was adopted to theorize the data. C. The authors have claimed on page 4 to have used another theoretical framework: This study was guided by the theoretical framework of symbolic interactionism, with the understanding that the symbols and meanings of a given phenomenon emerge from an individual’s interaction with society [13, 14]. C. The authors should decide which one to use, because they are not the same.

**Solution**: We understand the concern regarding the mention of symbolic interactionism and the framework for patient-centered access to health care as references for the study. We assert that symbolic interactionism was identified as the foundation for formulating the research topic and the principal framework of Grounded Theory. A conceptual framework for patient-centered access to healthcare has been identified as a reference for interpreting the data. We concur with the reviewer and have chosen to eliminate symbolic interactionism from the approaches to prevent ambiguity. This judgment is founded on a thorough methodology in our research, which is not fundamentally anchored in symbolic interactionism.

**Suggestion 4:** C. Now, reading the results, it becomes clear that the authors have followed the theoretical framework of patient-centered access to health care, which is ok. Then it gets confused by the fact that in the tables the authors present categories again, with their codes. It offers the reader a comprehensive look within the raw data, which is interesting. Yet, it causes some confusion. See, if you have the categories presented in the results (of the Abstract as 1) Ability to perceive; 2) Ability to seek; 3) Ability to reach; 4) Ability to pay, and 5) Ability to engage, and categories related to the service offered: 1) Approachability; 2) Acceptability; 3) Availability and accommodation; 4) Affordability; 5) Appropriateness. And then, when you read the results, you find on page 11: Table 2 presents Institutional Barriers of the Prison System, with its categories, subcategories, and illustrative statements.

**Solution:** We begin by affirming our agreement with the theoretical framework that highlights patient-centered access to health care in our analysis. We have excluded symbolic interactionism citation from our study's. Upon reviewing the correction from the reviewer and revisiting our paper, we recognize that we failed to elucidate the integration of the framework and categories. It may be a case of input confusion in our manuscript. To address this issue, we enhance specific aspects of the process and results sections, as detailed below. We emphasized that expression categories and subcategories, rather than codes, were utilized following the most recent change.

(a) The "Data Analysis" portion of the "Methodology" undergoes enhancements to elucidate the organization of categories in accordance with the selected theoretical framework: “The codes were grouped and linked into explanatory categories of the studied phenomenon according to the dimensions of access postulated on the Patient-Centered Access Conceptual Framework. [13, 14]. At this stage, gaps identified in the understanding of the phenomenon indicated questions that precipitated the need for additional interviews. Once theoretical saturation was achieved, the last stage of coding was conducted, wherein the categories organized according to the theoretical framework generated the final theorization [13, 15].” (lines 119 and 124).
(b) The "Results" section undergoes a new introduction: “Categories were formed aligning the meanings produced by prisoners during the interviews with the dimensions of the patient-centered access to healthcare conceptual framework [13, 14]. The theorization supported by theoretical reference allowed to organize the categories into two domains related to access for incarcerated men.

The first domain grouped patient abilities necessary to access health services. In our study, we named this theme “Prisoners’ Abilities in Accessing Oral Health Care in the Prison System”. The second assembled characteristics of health services related to access. This theme was named “Structural Barriers of the Prison System”. (lines 154 and 161).

(c) Table 1 presents the categories, subcategories, and illustrative statements of the theme Prisoners’ Abilities in Accessing Oral Health Care in the Prison System. (lines 162 and 163).
(d) Table 2 presents the categories, subcategories, and illustrative statements of the theme Institutional Barriers of the Prison System. (lines 204 and 206).

**Suggestion 5:** The C. See, for the reader, this comes at the difficult task to organize in his/her mind something that is ‘Institutional Barriers of the Prison System’ without knowing what it is, really. At least in my view, it seems that you have produced two Domains, or Themes – they can be used interchangeably [see Grounded Theory by Corbin and Strauss (Basics of qualitative research: Techniques and procedures for developing grounded theory, 3rd ed. Citation. Corbin, J., & Strauss, A. (2008).], and then the presentation should be organized under two Themes or Domains, being them (as the authors have manifested in the Abstract): … and analysis allowed the creation of the following ‘categories related to the patient’ … and then … ‘Structural Barriers of the Prison System’. They seem to me two domains, one based on the theoretical framework used, the last one based on the classical analysis that underscores the emerging of categories in Table 2.Therefore, such organization is demanded, so the reader can understand the Results and more, the way the theoretical framework has been used, and the way the Grounded Theory has been used to produce such results shall also be explained to avoid confusion.

**Solution:** We thank you for the feedback. We concur that enhancing the description of our analysis and findings was essential. We hope that improvements made to answer the last suggestion are enough to clarify these aspects. We are willing to implement further enhancements, if required.

**DISCUSSION**

**Suggestion 6:** Finally, I still think that the Themes, Categories and even Codes could be more thoroughly discussed in the context of the relevant literature, in the Discussion section. The authors bring interesting results with a scheme of coding and categorizing that resulted in a broad list of categories, even two Themes (in the hierarchy of Grounded Theory, one step below the creation of a Theory). Yet, the Discussion brings again a review of the literature, with no confrontation between the findings and the literature, except for the Micro-politics of Access, which is really an interesting finding and has been discussed. So, generally the Discussion follows a sequence of discussing the categories with the relevant literature, not necessarily all categories, but the most relevant indicated by the authors. I am not criticizing the review and the information brought about within the Discussion, which is fine. What I am discussing is the fact that there is no link between the Results and this dialogue that is expected in this section.

Therefore, I hope to see a deeper contextualization of the results, the empirical results and the relevant literature in the field, beyond the general and sometimes distant topics contained in the Discussion, at this point.

**Solution:** We are pleased with the positive points highlighted regarding our discussion, and we have engaged in making this section better integrated with the literature. In response to reviewer notes, we have carefully revised and restructured the “Discussion” section. We have sought to systematically and critically integrate the categories presented in the results. Following, we present the additional discussion inserted.

(a) One of the themes established in this study highlights characteristics of the prison system and their negative impacts on the access to dental care of incarcerated men. This outcome relates to overcrowding, and insufficient criminal police agents and dental surgeons. Therefore, there is a repressed demand within the prison system that undermines access. (lines 267 and 270).
(b) Structural restrictions to access oral health care found in our results have also been reported in other prisons, including those of high incomes countries. Evidence from a French study indicated that dental facilities in prisons exhibited considerable structural deficiencies, such as inadequately designed physical areas for dental practice, outdated equipment, and insufficient maintenance [24]. Moreover, challenges in inmate transportation and a lack of personnel limit dental surgeons to addressing only emergency cases [24]. Likewise, the United Kingdom, United States of America, Canada, and Australia presented deficiency of skilled practitioners, inadequate materials and equipment, and an absence of a structured care routine, which constitutes obstacles to dental treatment [25]. (lines 300 and 308).
(c) Our findings suggest that prisoners had the capacity to recognize their oral health issues. However, there is a lack of transparency and predictability regarding the mechanisms for accessing dental care, even when pain is perceived. In other studies [Bergum et al. (2024) and Zajmi et al. (2018)], incarcerated individuals recognize access to oral health care available in times of severe pain or obvious emergencies. Despite the differences, our and other results indicate a reactive nature of prisonal system, without planning of preventive or continuate care. (lines 321 and 326).
(d) The findings of this study suggest that access to dental care is constrained by healthcare professionals' prejudices against the incarcerated population, which undermines the legitimacy of inmates' needs. This results in a micro-policy of access that reflects the relevance of interpersonal relationships to access health services (irrespective of the real need for oral health). (lines 344 and 347).
(e) Brazil has a universal healthcare system that includes the provision of dental care to the prison population. However, the provision of health services in the prison system does not meet the demands for dental care [08]. Given the difficulty of obtain oral health care, our results showed that some inmates end up using private external services when they have ability to pay. This finding indicates the challenges of adequate financing the oral health services into the prison system and may be observed in other countries. In South Korea, prisoners are excluded from the public health insurance system and depend only on services provided by correctional facilities. Nonetheless, the demand for care exceeds the supply [43]. In the United States, which lacks a universal healthcare system, access to dental services is limited. Individuals with a history of incarceration are less likely to keep health insurance coverage. Consequently, efforts are required to enhance health insurance coverage to mitigate the health inequities seen by this population [44]. (lines 362 and 372).

We reiterate our gratitude for the valuable contribution, which certainly strengthened the quality of the manuscript. We remain at your disposal for further improvements and explanations.
