# Supplementary material for: Theorization regarding access to oral health care for Brazilian prisoners: A qualitative study
Source: PLoS One. 2025 Oct 31;20(10):e0335590. doi: 10.1371/journal.pone.0335590 (PMC12578342; doi:10.1371/journal.pone.0335590)
Supplement: Attachment — Submitted filename: Theorization regarding access to oral health care.docx [file pone.0335590.s003.docx]

This is the review of the manuscript “Theorization regarding access to oral health care for Brazilian prisoners: a qualitative study”_R2.

This time I feel very satisfied with the manuscript. All my questions have been answered, and the result is a much better manuscript, in my view. I therefore recommend publication.

Thanks for the opportunity to review this work for PLOS ONE.
