# Supplementary material for: Theorization regarding access to oral health care for Brazilian prisoners: A qualitative study
Source: PLoS One. 2025 Oct 31;20(10):e0335590. doi: 10.1371/journal.pone.0335590 (PMC12578342; doi:10.1371/journal.pone.0335590)
Supplement: Attachment — Submitted filename: Response_to_reviewers_auresp_3.docx [file pone.0335590.s005.docx]

**Dear Editor,**

We are pleased to have a new opportunity to further improve our manuscript. We conducted additional reviews according to the suggestions below:

**Suggestion 1**: *“In Lines 355-361 several strong claims are made which do not appear to be fully supported by the study, and references are not provided. To address this, please tone down all of the claims made in this paragraph, making it clear that your claims are speculative and not conclusions. For example, the first sentence could be rephrased as ‘This conduct may be shaped by ideological beliefs and ethical considerations grounded in a conservative perspective that the prison setting confines individuals who ought to forfeit all their rights, not merely their liberty’.”*

**Solution**: We agree with the reviewer's suggestion, and corrections were made as suggested, with additional text modifications in lines 356, 357, 360, and 362:

“This conduct may be shaped by ideological beliefs and ethical considerations grounded in a conservative perspective that the prison setting confines individuals who ought to forfeit all their rights, not merely their liberty. These beliefs could affect the dynamics between criminal police officers and inmates, as well as the operation of healthcare services. This scenario may have the potential to stigmatize the incarcerated population and rationalize the denial of rights, including oral health care, since prejudice diminishes the quality of health care and impedes the execution of governmental policies [40, 42].

**Suggestion 2**: *“In lines 379–380 you write “barriers to accessing oral health have been attributed by incarcerated men, mainly, to governmental negligence.’” This is a claim well supported by your data. In other places where the text makes claims about governmental negligence, you should make clear that this is the perception of your research participants, or else cite other evidence to support the claim. For example, in lines 344–346 you write, "The findings of this study suggest that access to dental care is constrained by healthcare professionals' prejudices against the incarcerated population, which undermines the legitimacy of inmates' needs." The methodology of this study cannot support this claim; the data relates to inmates’ experiences and perceptions of the care they receive. Please revise your submission to ensure your conclusions are supported by the results reported.”*

**Solution:** We now explicitly state in line 344 that our findings stem from the inmates’ perspective, and we also reviewed the phrasing in line 346:

“The perspective of the inmates presented in our results indicates access to dental care may be constrained by healthcare professionals' prejudices against the incarcerated population, which undermines the legitimacy of inmates' needs.”

We again reiterate our gratitude for the valuable contributions and for the approval of the manuscript. We remain at your disposal for further refinements and explanations.
